# BMI z-score in obese children is a poor predictor of adiposity changes over time

**DOI:** 10.1186/s12887-018-1160-5

**Published:** 2018-06-08

**Authors:** Cassandra Vanderwall, Jens Eickhoff, R. Randall Clark, Aaron L. Carrel

**Affiliations:** 1 University of Wisconsin, UW Health- University Hospital, 600 Highland Ave, Madison, WI 53792 USA; 2 UW Health Department of Sports Medicine, Madison, WI USA

**Keywords:** Body mass index Z-score, Childhood obesity, Dual energy X-ray absorptiometry, Body composition

## Abstract

**Background:**

The age and sex standardized body mass index (BMIz) is a simple and widely utilized screening tool for obesity in children and adolescents. The purpose of this study was to evaluate the relationship between the BMIz trajectory versus the percent body fat (%FAT) trajectory, and if BMIz could predict significant changes in %FAT in a sample of obese children and adolescents.

**Methods:**

In this longitudinal observational study, body composition was measured by dual energy x-ray absorptiometry (DXA) in obese children within a multidisciplinary pediatric fitness clinic at an academic medical center over a 3-year time period. Regression analyses were conducted to evaluate the association between changes in BMIz and changes in %FAT.

**Results:**

Baseline assessment was obtained from 515 participants. The reduction observed in BMIz (2.20 to 2.08, *p* < 0.0001) correlated with the reduction in %FAT (38.5 to 35.8%, *p* < 0.05) in the first two years. The overall correlation between the slope in BMIz reduction versus %FAT reduction was moderate (*r* = 0.36, p < 0.0001) over the 3-year follow-up period. The sensitivity of BMIz changes for predicting a decrease in %FAT was acceptable (70, 95% CI: 61–78%), however the specificity was poor (42, 95% CI: 31–54%).

**Conclusions:**

These findings advance the understanding of the utility and limitations of BMIz in children and adolescents. While BMIz may be sensitive to changes in adiposity, it is a weak predictor of these changes in total body fat (%FAT) due to the poor specificity. Therefore, clinicians must exercise caution when monitoring changes in a growing child’s body composition to avoid misclassifying or missing substantial change when utilizing BMIz alone.

## Background

The body mass index (BMI) is the most widely available tool for monitoring progress in the campaign against obesity [1]. The BMI correlates well enough with direct measures of adiposity to support its use across age groups [1]. However, it is a flawed proxy with several limitations due to variability in the association between BMI and total percent body fat (%FAT), risk of chronic disease and long-term outcomes of obesity-related disease.

Measures of overweight and obesity are typically generated via population-level statistics and use a variety of reference data sets for BMI which causes there to be differences in both metrics and terminology [2]. This discrepancy in definitions does not mean that the screening tool is not valuable, but rather that additional evidence is needed to support its utility. The BMI varies with age in children and thus BMI values are compared with age- and sex-specific references. Also, due to these variations a normalizing transformation is necessary into a Z-score. The Centers for Disease Control has defined obesity as a Z-score > 1.64 and overweight as a Z-score > 1.04 It is recognized that z-scores were not designed for longitudinal data analysis; despite this, many clinicians believe that a change in BMIz represent a change in adiposity [3].

Anthropometric measures and common clinical body composition tools, including waist circumference, skinfolds, bio-electrical impedance analysis (BIA), air displacement plethysmography (ADP) hydrodensitometry, and body weight, used to assess and monitor body composition are subject to error and have the potential to misclassify and inaccurately track statistically significant changes in body composition (poor specificity). However, due to ease of acquisition, the most widely used clinical outcome variable is BMI, and standardized for age and sex, as BMIz. Previous reports, including ours evaluated the strength of how well BMIz predicts adiposity in children [4, 5]. However, whether the longitudinal change in BMIz over time predicts a true change in adiposity is unknown.

Appropriate diagnosis of childhood obesity is critical to an appropriate clinical and public health response to the obesity epidemic [6]. Research of more sophisticated measures for assessing adiposity in children and adolescents is needed [6, 7]. At present, it is unclear whether BMI can track changes from interventions designed to improve body composition in overweight and obese children and adolescents. The purpose of this investigation is to evaluate the relationship between the BMIz trajectory versus the percent body fat (%FAT) trajectory, and determine if change in BMIz can effectively track real changes in %FAT in obese children participating in an intervention designed to improve body composition.

## Methods

All subjects were obese boys and girls (BMI-for-age ≥ 95 percentile) ages 4 to 18 years evaluated as part of their routine clinical care at a multidisciplinary weight management program within an academic medical center. Anthropometric and body composition measurements were collected in accordance with previously published methods [4, 5, 8–10]. Study procedures were approved by the Health Sciences Human Subjects Committee at the University of Wisconsin- Madison.

All baseline characteristics were summarized in terms of means (SD) or frequencies and percentages. Linear mixed effects modeling with subject specific random effects was conducted to evaluate changes in BMIz and %FAT and the association between BMIz and %FAT over the 3-year follow-up period. Residual plots and normal probability plots were examined to verify the model assumptions. Furthermore, subject-specific regression analyses of %FAT and BMIz on time were conducted. A subject specific decrease in %FAT or BMIz over the 3-year follow-up period was defined as a negative slope for corresponding regression model. A minimum sample size of 60 subjects was required to detect an anticipated moderate correlation of at least 0.4 between decrease in %FAT and the corresponding decrease in in BMIz at the two-sided 0.05 significance level. Sensitivity and specificity of BMIz status (decrease versus non-decrease) for predicting a decrease in %FAT were calculated and reported along with the corresponding 95% confidence intervals. SAS software version 9.4 (SAS Institute Inc., Cary NC) was utilized for all statistical analyses; reported *P*-values are two-sided and statistical significance was defined as *P* < 0.05.

## Results

**Fig. 1:**
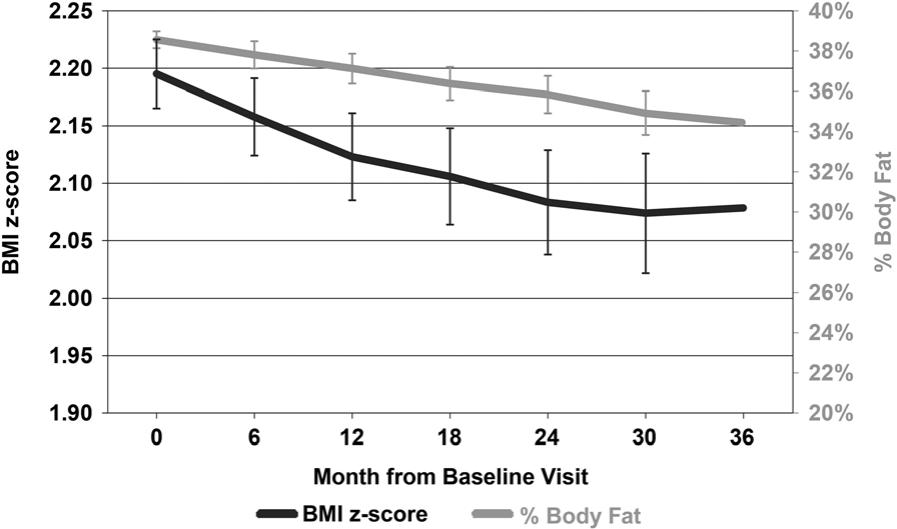
Changes in BMI z-score (BMIz) and total percent body fat (%FAT) over the three-year follow-up period in obese children and adolescents (*n* = 511)

Subjects were 515 obese boys and girls (49% male) with a mean (SD) age of 11.5 (3.3) years, BMI of 29.6 (6.3) and BMIz of 2.2 (0.5). All patients were deemed obese based on their BMI-for-age percentile. Mean criterion body composition values were a total fat mass (TFM) of 32.9 (14.2) kg and %FAT of 38.8% (5.6) in the sample (Table 1, Fig. 1). Subjects with a baseline visit and at least one follow-up visit were included in the analysis.

**Table 1:** Characteristics of the study population (*N* = 515)

	Mean (SD)
Age (years)	11.5 (3.3)
BMI	29.6 (6.3)
BMI z-score	2.21 (0.5)
BMI percentile	97.1 (3.4)
Weight (kg)	65.6 (27.4)
Height (cm)	144.2 (23.3)
Total Fat Mass (kg)	32.9 (14.2)
Percent Body Fat	38.8 (5.6)
VO2 Max	32.7 (5.8)
	N (%)
Sex (Male)	252 (49)
Race-Ethnicity	
Non-Hispanic White	374 (73)
Non-Hispanic Black	57 (11)
Hispanic	129 (25)
Asian	25 (5)
American Indian	34 (7)

**Fig. 2:**
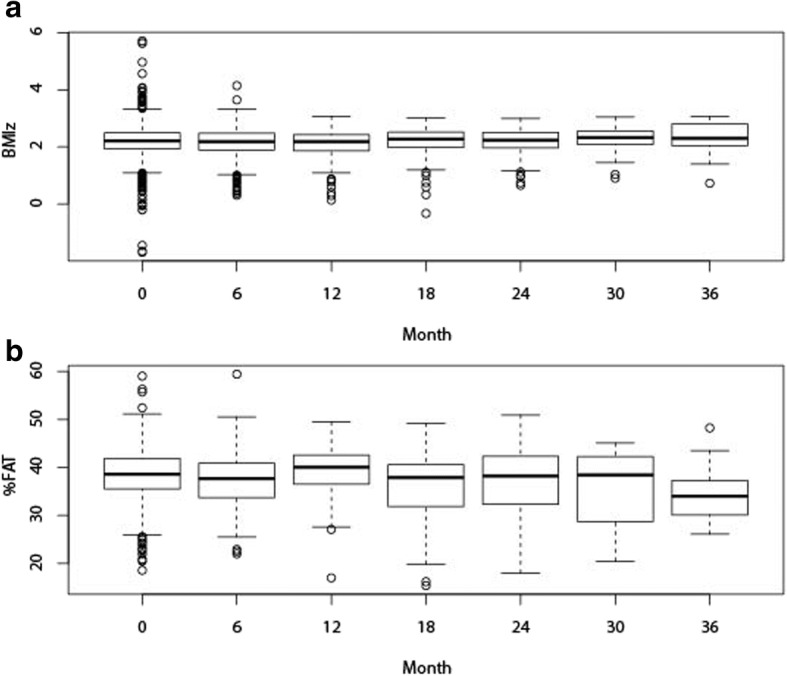
Box and Whisker plots of distributions of changes in (**a**) BMI z-score (BMIz) and (**b**) total percent body fat (%FAT)

There was a significant decrease in the mean BMIz from baseline (2.20, 95% CI: 2.17–2.23) to the end of the 3-year follow-up period (2.08, 95% CI: 2.02–2.14, *p* < 0.0001) (Table 2, Fig. 1). There was also a modest, significant decrease in the mean %FAT from baseline (38.5, 95% CI: 38.1–39.0%) to the end of the follow-up period (34.5, 95% CI: 33.1–35.8%, *p* < 0.05) (Table 2, Fig. 1). The distribution of changes in %FAT and BMIz over time are shown in Fig. 2. The overall correlation between the BMIz versus %FAT slope over time was moderate (*r* = 0.36, *p* < 0.0001) and varied over the 3-year follow-up period (Table 3, Fig. 1). Interestingly, the correlation was stronger between the first and second year after baseline (*r* = 0.63, *p* < 0.0001).

**Table 2:** BMI z-score and % Body Fat from month 0 to month 36

Month	N	BMI z-score		% Body Fat	
		Mean	95% CI	Mean	95% CI
0 (Baseline)	515	2.20	2.17–2.23	38.5%	38.1–39.0%
6	452	2.16	2.12–2.19	37.8%	37.1–38.5%
12	212	2.12	2.09–2.16	37.1%	36.4–37.9%
18	132	2.11	2.06–2.15	36.4%	35.6–37.2%
24	98	2.08	2.04–2.13	35.8%	34.9–36.8%
30	65	2.07	2.02–2.13	34.9%	33.8–36.0%
36	46	2.08	2.02–2.14	34.5%	33.1–35.8%

**Table 3:** Correlation between slope of BMIz changes versus slope of %FAT changes

	Correlation	95% CI	*p*-value
Month 0 to Month 36	0.36	0.23–0.47	< 0.0001
Month 0 to Month 12	0.31	0.16–0.46	0.0001
Month 12 to Month 24	0.63	0.42–0.77	< 0.0001
Month 24 to Month 36	0.49	0.06–0.77	0.0231

Furthermore, in order to further evaluate the role of BMIz changes as a potential predictor for changes in %FAT, sensitivity and specificity of BMIz changes for predicting a decrease or increase in %FAT were calculated. The sensitivity of BMIz changes over the 3-year follow-up period for predicting a decrease in %FAT was acceptable (70%). However, the corresponding specificity of BMIz changes was poor (42%) (Table 4), confirming that a BMIz change is not a strong predictor of the corresponding change in %FAT. For example, if a subject had an overall decrease in %Fat over the follow-up period, then there was a 70% chance that the subjects had also an overall decrease in %BMIz, however, if a subject had an overall increase in %Fat, then there was only a 42% chance that the subject had also an overall increase in BMIz over the follow-up period. The corresponding positive and negative predictive values were 67 and 45%, respectively (Table 4). Furthermore, the area under the receiver operating curve (ROC) when evaluating the association of BMIz changes for predictive a decrease in %FAT over the 3-year follow-up period was only 0.64 (95% CI: 0.55–0.72), confirming that BMIz change not a strong predictor for a corresponding change in %FAT.

**Table 4:** Sensitivity, specificity, positive predictive value, and negative predictive value (95% CI) of BMI z-score (BMIz) decrease for predicting a decrease in Percent Body Fat (%FAT)

Month	N	K	Sensitivity	Specificity	Positive predictive value	Negative predictive value
0–36	118	113	70% (61–78%)	42% (31–54%)	67% (58–75%)	45% (33–57%)
0–12	81	77	68% (56–77%)	31% (19–46%)	64% (53–74%)	46% (30–64%)
12–24	27	23	83% (63–93%)	60% (39–78%)	70% (52–84%)	75% (51–90%)
24–36	10	13	62% (36–82%)	60% (23–88%)	80% (49–94%)	38% (14–69%)

N: Number of subjects with a decrease in BMI z-score (BMIz)

K: Number of subjects with a decrease in Percent Body Fat (%FAT)

## Discussion

While BMI is a simple and widely used screening tool for obesity, its ability to assess change in body composition over time is unknown. The index provides a common foundation for comparing individuals [11], but BMI cannot differentiate between fat and muscle. Thus, BMI has utility for screening and epidemiologic research however; there are limitations and increased risk for misclassifying growing children when using BMI and BMIz alone to define overweight and obesity [7]. This limitation may be due to the strong interaction between age and %FAT, where in children younger than 9 years, the BMIz is a weak predictor for both total fat mass and %FAT but BMIz is a stronger predictor of TFM in youth over the age of 9 years [4]. These results have strong implications for the use and reliance on the BMI for screening and monitoring weight-related changes in overweight and obese youth.

This study evaluated the relationship between BMIz and %FAT as determined by DXA before and after an intervention designed to improve body composition. The results confirmed that changes in %FAT cannot be predicted accurately by changes in BMIz alone. Likewise, Freedman (2009) examined if overweight and obese youth with excessive adiposity were also among those classified with a BMI-for-Age ≥ 85th percentile [12]. They found that nearly 77% of the children who had an obese BMI-for-Age (≥ 95th) percentile were classified as having elevated adiposity. However, those with a BMI-for-Age between the 85th and 94th percentiles (overweight) were variable with about 50% having moderate adiposity, 30% normal adiposity (median 10%) and only 20% have an elevated body fatness. They concluded that BMI is an appropriate screening tool to identify children who require further evaluation but is not a diagnostic method for assessing adiposity. The present analysis supports Freedman’s findings in that BMIz maintains an appropriate sensitivity to changes in adiposity over time but is unreliable due to poor specificity in predicting changes in %FAT [12].

While other studies have demonstrated BMI to have greater correlation with adiposity, our results discourage the use of BMI and BMIz for longitudinal monitoring of changes in adiposity among obese children and adolescents. There is growing evidence that the correlations between changes in BMI, BMIz and BMI-for-age percentiles and changes in adiposity (TFM, %FAT) are significantly lower than previously thought [13]. Accurately measuring changes in adiposity during childhood has vital implications for clinical management and treatment.

A strength of the current study was the large cohort (*n* = 515) of obese children whom were monitored over an extended period of time (3 years) with use of DXA. The present assessment is novel because it 1) utilizes DXA to establish the degree of adiposity in these subjects to evaluate these relationships in a large cohort (n = 515) of obese children whom were monitored over an extended period of time (3 years), 2) identifies the poor positive predictive value of BMIz relative to %FAT over time, and 3) supports our previous identification of the non-predictive nature of BMIz relative to TFM in younger children (4–9 years). A limitation of the present observational study is the large percentage of subjects loss to follow-up. The small sample sizes at follow-up assessment resulted in wide confidence intervals when evaluating the diagnostic properties of BMIz changes when predicting changes in %FAT. The primary objective of this study was not to evaluate changes from the baseline to the 2 or 3-year follow-up visit, but rather to evaluate the association between the BMI z-score (BMIz) and body fat percent (%FAT) trajectories over a 36-month follow-up period. Data from all initial patients (*N* = 515) at every visit was not required to provide robust and unbiased estimates of the trajectories. The trajectories were quantified by calculation and the regression slopes change linearly over time. While the prediction error at a particular time point is affected by the sample size at each time point, the standard error of the slope parameter estimate is constant. Therefore, we believe the present analysis is still accurate and pertinent to better understanding the relationship between BMIz and %FAT.

Another limitation and area of future investigation would be to identify the difference in correlations or associations by race-ethnicity, age, and pubertal status. Presently, no data was collected to classify pubertal status for the current study sample. It is well-established adiposity (%FAT) varies according to biological sex, maturation, pubertal status and race-ethnicity. Early maturation has been observed in overweight and obese girls, which has directly implications upon adiposity, visceral fat stores and overall body composition often leading to greater fat, lean and bone mass, although these vary by race-ethnicity [14, 15]. Overweight and obese status within boys may delay maturation and thus negatively impact body composition, inclusive of lean, fat and bone mass. The evidence regarding the exact mechanism and impact of maturation upon body composition remains mixed and is an opportunity for future investigations.

Finally, practicality and feasibility limit the use of DXA for monitoring changes in adiposity in obese youth and thus most clinicians utilize BMI with the supposition that an elevated BMI is equivalent to excess adiposity [16–19]. However, BMI is not indicative of the degree of adiposity in younger children [4, 5, 20, 21].

## Conclusion

This study demonstrated that changes in BMIz were not predictive of statistically significant changes in %FAT in a sample of 515 obese boys and girls evaluated by DXA over 3-year period. The sensitivity of BMIz changes for predicting a decrease in %FAT was 70%. However, the specificity of BMIz for predicting a decrease in %FAT was only 42%. This poor specificity indicates that changes in BMIz were not predictive of changes in %FAT in this sample under the conditions of the study. Based on these results we recommend that clinicians exercise caution when monitoring changes in body composition to avoid misclassifying or missing a statically significant change in %FAT in the obese pediatric population when utilizing BMI alone [7, 18, 19, 22, 23]. Thus, while BMIz may be a screening tool for obesity, it was not an accurate tool for monitoring change in adiposity in this sample of obese children.

## Abbreviations

%FAT: Total percent body fat
BMI: Body mass index
BMIz: Body mass index z-score
DXA: Dual energy x-ray absorptiometry
TFM: Total fat mass
